# Could Naringenin Participate as a Regulator of Obesity and Satiety?

**DOI:** 10.3390/molecules28031450

**Published:** 2023-02-02

**Authors:** Gabriela López-Almada, J. Abraham Domínguez-Avila, María Esther Mejía-León, Maribel Robles-Sánchez, Gustavo A. González-Aguilar, Norma Julieta Salazar-López

**Affiliations:** 1 Facultad de Medicina de Mexicali, Universidad Autónoma de Baja California, Dr. Humberto Torres Sanginés S/N, Centro Cívico, Mexicali 21000, BN, Mexico; 2 CONACYT—Centro de Investigación en Alimentación y Desarrollo A. C., Carretera Gustavo Enrique Astiazarán Rosas No. 46, Col. La Victoria, Hermosillo 83304, SO, Mexico; 3 Departamento de Investigación y Posgrado en Alimentos, Universidad de Sonora, Blvd. Luis Encinas y Rosales, Col. Centro, Hermosillo 83000, SO, Mexico; 4 Centro de Investigación en Alimentación y Desarrollo A. C., Carretera Gustavo Enrique Astiazarán Rosas No. 46, Col. La Victoria, Hermosillo 83304, SO, Mexico

**Keywords:** overweight, satiety, naringenin, enterohormones, dyslipidemia, phenolic compounds

## Abstract

Obesity is a serious health problem worldwide, since it is associated with multiple metabolic disorders and complications such as cardiovascular disease, type 2 diabetes, fatty liver disease and overall metabolic dysfunction. Dysregulation of the hunger–satiety pathway, which includes alterations of central and peripheral signaling, explains some forms of obesity by favoring hyperphagia and weight gain. The present work comprehensively summarizes the mechanisms by which naringenin (NAR), a predominant flavanone in citrus fruits, could modulate the main pathways associated with the development of obesity and some of its comorbidities, such as oxidative stress (OS), inflammation, insulin resistance (IR) and dyslipidemia, as well as the role of NAR in modulating the secretion of enterohormones of the satiety pathway and its possible antiobesogenic effect. The results of multiple in vitro and in vivo studies have shown that NAR has various potentially modulatory biological effects against obesity by countering IR, inflammation, OS, macrophage infiltration, dyslipidemia, hepatic steatosis, and adipose deposition. Likewise, NAR is capable of modulating peptides or peripheral hormones directly associated with the hunger–satiety pathway, such as ghrelin, cholecystokinin, insulin, adiponectin and leptin. The evidence supports the use of NAR as a promising alternative to prevent overweight and obesity.

## 1. Introduction

Approximately one third of the world’s population is currently classified as overweight or obese [1]. The World Health Organization (WHO) defines obesity according to a body mass index (BMI) ≥ 30 kg/m^2^ and an abnormal or excessive accumulation of adipose tissue, which can be detrimental to health due to its association with multiple metabolic disorders [2]. Abdominal obesity is an important risk factor for the development of metabolic syndrome (MetS) which, through hypertension, hyperglycemia and dyslipidemia, increases the risk of cardiometabolic complications such as cardiovascular disease, type 2 diabetes mellitus (T2DM) and metabolic-associated fatty liver disease (MAFLD) [3].

The causes of obesity are complex and multifactorial, and include unbalanced caloric intake, environmental factors, reduced physical activity and genetic factors, among many others [4]. Improperly regulated pathways of hunger and satiety are particularly highlighted, a condition that leads to hyperphagia and long-term positive energy balance that culminates in weight gain and obesity [5].

Satiety is considered a feeling of fullness after eating that suppresses hunger and inhibits the action of eating between meals [6]. The regulation of the beginning and cessation of food intake, as well as the interval between meals, is mediated by a complex and structured control mechanism that integrates peripheral signals in the central nervous system into hunger/satiety, actions related to food intake and systemic energy metabolism [7]. In this regard, previous studies have reported that certain dietary compounds are capable of modulating some hunger–satiety mediators, which could directly impact adipose deposition and, therefore, body weight [5,8].

Phenolic compounds are secondary plant metabolites that are found naturally in foods such as fruits, vegetables, cereals, legumes, dark chocolate, coffee, tea and wine, among others [9]. Previous research has shown that diets rich in phenolic compounds offer beneficial effects to human health, by aiding in weight control and obesity [9,10]. For example, some flavonoids are known to favorably alter the hunger–satiety pathway by modulating some key peptides [5,11], which, in turn, can prevent the development of chronic non-communicable diseases (NCDs), such as those associated with obesity [12,13].

In particular, naringenin (NAR) is a flavonoid that can be found in the pulp and peel of *Citrus* species (lemon, grapefruit, orange, tangerine, bergamot and others), as well as in other fruits such as figs and tomato peel [14]. Flavonoids have been extensively researched, and NAR exerts bioactivities closely related to weight control and obesity; thus, there is significant interest in its continued study. For example, many in vivo studies report NAR’s ability to prevent weight gain or induce weight loss [15,16,17,18,19]. Consistent with these findings, Namkhah et al. [20], through a randomized, double-blind, placebo-controlled clinical trial, showed that daily NAR supplementation for 4 weeks in overweight/obese patients with non-alcoholic fatty liver disease (NAFLD) significantly reduced their weight and BMI, improved their lipid profile (reduced serum TG and LDL and increased HDL), liver steatosis and fibrosis. However, some data remain to be elucidated, such as determining if the apparently antiobesogenic effect of NAR is due to it modulating the participants’ hunger/satiety signals, including peripheral regulation through a mechanistic insight or evaluation. The present review comprehensively describes the mechanisms by which NAR could modulate the main pathways associated with obesity, including OS, inflammation, IR, dyslipidemia and regulation of the hunger–satiety pathway.

## 2. General Description of Naringenin

**Figure 1:**
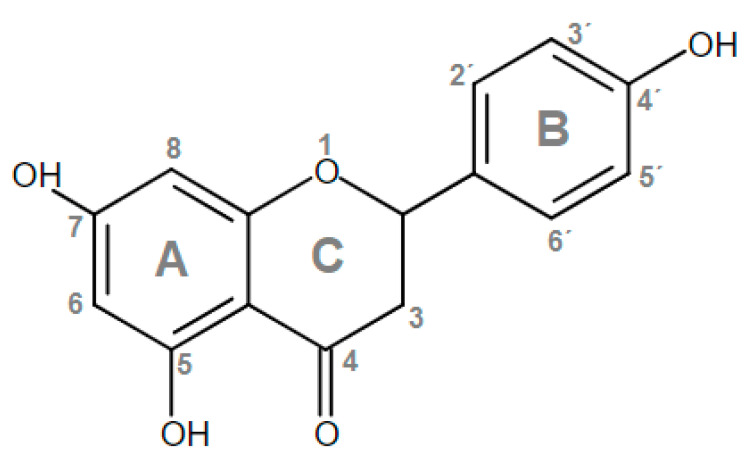
Chemical structure of naringenin (NAR).

The chemical structure of phenolic compounds is made up of one or more aromatic rings with at least one hydroxyl group attached to them (monophenols or polyphenols, respectively). Flavonoids are the most abundant class of phenolic compounds, and can be further subdivided into 6 subgroups, namely, anthocyanidins, flavonols, flavanones, flavanols, flavones, and isoflavones [21]. NAR (4′,5,7-trihydroxyflavanone) is a flavanone (Figure 1), that is characterized by the presence of three hydroxyl groups on rings A and B. These chemical groups and their localization play a central role in its antioxidant effect, since they confer it significant H^+/−^ or electron-transfer potential that can neutralize free radicals [22].

NAR is the aglycone form of naringin (4′,5,7-trihydroxyflavanone-7-rhamnoglucoside), which is a neohesperidoside and one of the main flavanone glycosides, and is, therefore, generated from the hydrolysis of naringin [23]. NAR is also considered the active metabolite of naringin, and its release has been considered the limiting step for its enteric absorption [24]. After oral intake, naringin and NAR are absorbed in the small intestine [24,25]; in vivo data suggest that NAR is mainly absorbed in the duodenum, which coincides with extensive metabolism reported to occur therein, as compared with other segments of the small intestine [26]. The main absorption mechanism appears to be passive diffusion [27] and active transport mediated by P-glycoprotein and Mrp1 proteins (multidrug resistance protein 1) [27,28].

After NAR enters systemic circulation, in either its free form or its metabolites, it is distributed to tissues. The predominant circulating metabolites have been identified as various glucuronides while, in tissues, free NAR and naringenin-7-*O*-sulfate are the predominant forms [29]. Although NAR is metabolized in vivo, to the best of our knowledge, the effects of its isolated metabolites have not been investigated on obesity; the present work, therefore, focuses on the effects of NAR, without differentiating between its metabolites.

## 3. Effects of Naringenin against the Various Components of Obesity

An abnormal and excessive accumulation of adipose tissue is a risk factor for the development of IR that precedes T2DM, and possibly MetS and numerous other NCDs and their comorbidities [24]. Adipose tissue hypertrophy and its dysregulation as an endocrine organ leads to abnormal secretion of free fatty acids, adipokines and proinflammatory cytokines, which in turn attract immune cells that increase damage in a positive feedback loop, while also promoting chronic low-grade inflammation and OS [25]. Obesity is, therefore, a physiologically complex disease, with multiple aspects that must be collectively addressed.

This section discusses reported effects of NAR on the aforementioned components of obesity.

### 3.1. Oxidative Stress (OS)

OS refers to an unbalanced redox state, in which the cell’s antioxidant defense mechanisms are insufficient against free radicals (FR), reactive oxygen species (ROS) and/or reactive nitrogen species (RNS). Normal levels of FR play an important role in modulating cellular and physiological processes; however, higher concentrations cause cell damage by attacking macromolecules and organelles, further amplifying FR production and compromising the organism’s homeostasis [30]. OS has been reported as a factor in the development and progression of various metabolic diseases, including T2DM, MetS, fatty liver disease, among others, which emphasizes the need of addressing it as part of a comprehensive obesity treatment [31].

To counter OS and maintain an adequate redox balance, mammalian organisms have an antioxidant defense system made up of enzymes and small molecules, with the ability to eliminate and/or prevent the formation of FR, ROS and RNS. Exogenous antioxidants are also important, particularly when the endogenous ones are overwhelmed [32]. Flavonoids are characterized by their antioxidant activity [22], for example, NAR neutralizes FR and prevents their formation by chelating metal ions (mainly copper and iron) that catalyze their synthesis. These bioactivities are dependent on its chemical structure, in particular, hydroxyl groups in position 4′ of ring B and in position 5 and 7 in ring A, as well as a carbonyl group in position 4 of ring C [33]. Among them, the 4′ hydroxyl group in ring B has been proposed as the most likely site of hydrogen atom transfer to eliminate FR [34].

In addition to exerting a direct antioxidant activity, NAR modulates the antioxidant system by promoting gene expression and enzymatic activity of the endogenous system [35]. It can also interact with biological membranes, including the cell membrane and those of organelles, and alter their structure, hydration, fluidity and permeability [36,37]. Such actions on lipid membranes contribute to the antioxidant effects of NAR by minimizing interactions between FR and lipids [35]. This has been confirmed in a rat model of myocardial ischemia-reperfusion, where NAR dose-dependently decreased malondialdehyde (MDA) levels, a product and biomarker of lipoperoxidation. The chelating capacity of NAR further contributes to the prevention of cell death by iron-dependent lipoperoxidation (ferroptosis) [38].

NAR also modulates the nuclear factor erythroid 2-related factor 2 (Nrf2) [39], a transcription factor that is a main regulator of the expression of antioxidant enzymes that respond to OS, through the Nrf2-Keap1-ARE pathway. Other studies have shown that NAR has an effect on the regulation of heme oxygenase 1 (HO-1), an inducible microsomal enzyme upstream of the Nrf2 axis. This has further increased interest on NAR, since it has been recognized as a novel molecule with pleiotropic effect, according to its ability to exert multiple pharmacological activities, since it has been shown to have antioxidant effects due to ROS elimination through bilirubin [40] and a neuroprotective effect in vitro [41].

NAR also exerts antioxidant effects through the modulation of the specific signaling pathways of the OS regulator Sirtuin 1 (SIRT1) [42], decreased activation of NADPH oxidase (which generates the superoxide anion) [43], and increased activity of superoxide dismutase (SOD), catalase (CAT), glutathione peroxidase (GPx) and glutathione (GSH) [33,44]. In vivo models show that NAR decreased plasma oxidant molecules and lipid peroxidation markers (nitric oxide and MDA), while stimulating the antioxidant system by significantly increasing SOD and GSH levels [19]. In adipose tissue, it increased glutathione reductase (GR) and SOD mRNA expression [45]. Hepatic effects have also been reported, for example, NAR decreased NADPH oxidase protein and MDA levels, while increasing SOD, CAT and GSH, with a tendency to a dose-dependent effect on the antioxidant system [42].

**Figure 2:**
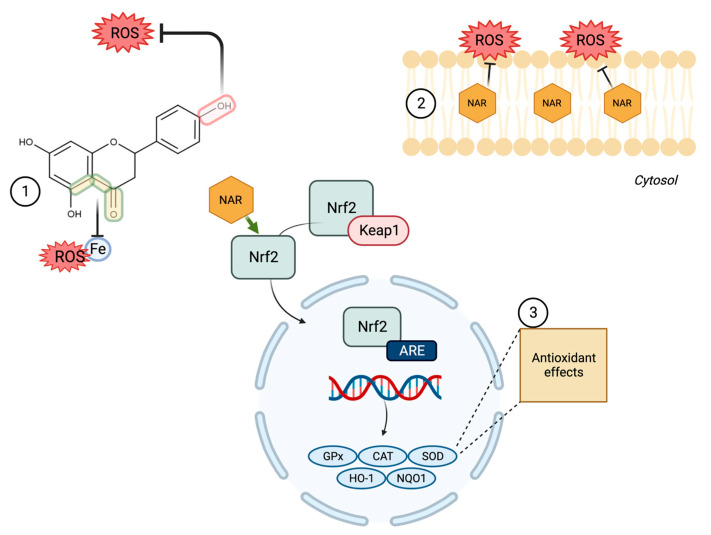
Main antioxidant mechanisms of NAR. (**1**) stabilizing free radicals (FR) due to transfer of hydrogen atoms and chelating metal ions. (**2**) Protecting lipid membranes by influencing their structure, hydration and fluidity, minimizing interactions between FR and lipids. (**3**) Modulating the Nrf2-Keap1-ARE pathway to induce gene transcription of the endogenous antioxidant system.

It has been suggested that the antioxidant effects of NAR are exerted in such a way that it acts to neutralize ROS as the first line of defense, and subsequently induces and dose-dependently activates the Nrf2-mediated antioxidant defense systems [46] (Figure 2). Because of this, NAR may be useful in diseases where OS is part of its pathophysiology, where its ability to stabilize FR, chelating potential, influence on lipid membranes and gene regulating effects are desirable; furthermore, it could synergize with other compounds, such as quercetin [47].

### 3.2. Inflammation

Obesity is associated with adipose tissue hypertrophy and changes that initiate an inflammatory process in adipocytes. Some of the mechanisms that regulate inflammation in this tissue include endoplasmic reticulum stress, hypoxia, free fatty acids and the recruitment of immune cells such as natural killer (NK) cells, neutrophils and macrophages in their proinflammatory phenotype [48]. Immune cells have been shown to be a source of proinflammatory cytokines such as tumor necrosis factor-alpha (TNFα), interleukin (IL)-1β, monocyte chemoattractant protein 1 (MCP-1), IL-4, IL-5, IL-6 and IL-12. These amplify inflammation by recruiting other immune cells and stimulating local proliferation of macrophages and their maintenance, eventually triggering adipose tissue dysfunction and promoting complications such as IR and glucose intolerance [49]. Such a chronic low-grade inflammation is characteristic of obesity; countering it can mitigate further development of associated NCDs.

NAR has been shown to modulate various inflammatory biomarkers in vivo. In obese mice fed a high-fat diet (HFD), it can inhibit neutrophil and macrophage infiltration in adipose tissue, as well as inhibiting the expression and secretion of proinflammatory and chemotactic cytokines such as MCP-1, MCP-3, but not the expression of macrophage inflammatory protein-2 (MIP-2) [50,51]. NAR can also decrease interleukins IL-6 and IL1A in adipose tissue, as well as IL-1B, IL-6 and TNF-α in serum and skeletal muscle [16,45,50]. The potential mechanism may be decreased mRNA expression of these cytokines in adipose tissue [45,50], suggesting a protective anti-inflammatory effect.

### 3.3. Insulin Resistance

Insulin is synthesized and secreted by β-cells of the pancreas in response to food intake, particularly carbohydrates. The critical role of insulin in glucose and energy homeostasis through regulating central and peripheral pathways is well known [52]. Insulin exerts an anorexigenic effect associated with weight loss, which has become documented when administered intracerebroventricularly or intranasally [53,54].

Obesity-associated IR may be due to the aforementioned adipose inflammation. Immune cells in adipose tissue induce macrophages into a proinflammatory phenotype and promote cytokine secretion, some of which can promote IR [48]. For example, cytokines such as TNFα and IL-6 induce the activation of IκB kinase β (IKKβ) and Jun N-terminal kinase (JNK1) signaling pathways [55], leading to increased phosphorylation of insulin receptor substrate 1 (IRS1), which decreases downstream insulin signaling [56]. It is also known that increased IL-6 hinders phosphorylation of the insulin receptor and IRS1, by inducing the expression of SOCS-3 [57]. Because IR may progress into T2DM, when NAR regulates IL-6 or other proinflammatory cytokines, a dual anti-inflammatory and antidiabetic effect is, therefore, exerted.

Bhattacharya et al. [58] analyzed the ability of NAR to promote glucose-dependent insulin secretion in vitro. It was found that NAR was capable of dose-dependently increasing insulin secretion under hyperglycemic conditions by up to 74.4% (acutely) or 33.3% (chronically) (*p* < 0.001; at concentrations of 10^−^⁶ M). It was also able to increase the gene expression of Ins1 and Ins2, glucose transporter (GLUT2) and glucokinase (GCK), while also promoting insulin signaling.

Supplementing a high-fat and cholesterol diet with 3% NAR was shown to improve glycemic parameters in a murine model after six weeks of dietary intervention [18]. NAR also decreases fasting glycemia by up to 37%, plasma insulin by 50% and improves HOMA-IR [16,17,18,45]. Li et al. [16] found that, in a mice strain genetically predisposed to diabetes, supplementing their diet with 100 mg NAR/kg diet improves, but does not normalize, glycemic parameters when compared with the parental non-diabetic strain. The evidence, therefore, suggests that NAR can improve some metabolic parameters in diabetic organisms and prevent its development in healthy ones.

Some reported mechanisms by which NAR decreases IR include downregulating the expression of proteins involved in endoplasmic reticulum stress [59]. Upregulating transcription and expression of PPAR-γ and GLUT4 has also been described [60]. Li et al. [16] reported that NAR increases GLUT4 membrane translocation in AMPK-dependent insulin resistant cells induced by TNF-α in vitro (C2C12 mouse myoblasts), while simultaneously suppressing the effects of TNF-α associated with the development of IR. This suggests that NAR may exert some regulatory effects on insulin signaling.

### 3.4. Dyslipidemia

Obesity is usually accompanied by dyslipidemias, i.e., alterations in lipid metabolism, such as hypercholesterolemia, hypertriglyceridemia, increased LDL-c, decreased HDL-c and increased circulating free fatty acids [61,62]. These complications increase the individual’s cardiometabolic risk; thus, addressing them is often carried out as part of an obesity treatment [63].

NAR has shown lipid-normalizing effects such as reducing plasma triglycerides (TG) and cholesterol by 46 and 23%, respectively, when LDLR-/- mice, a strain used for the study of atherogenesis and dyslipidemias, consumed a standard diet supplemented with 3% NAR [17]. This is in line with the results of Hua et al. [42], who reported that the administration of NAR (100 and 200 mg/kg) to apolipoprotein E-deficient mice (APOE-/-), significantly reduced serum cholesterol, TG and LDL-c, with a stronger effect with the higher dose. Interestingly, the 200 mg/kg dose prevented the decrease in HDL-c (*p* < 0.05), whereas TG concentration was normalized to levels similar to those of the wild strain. The mechanisms proposed include decreased de novo lipogenesis by downregulating hepatic mRNA of FASN and SCD1 (stearoyl-CoA desaturase 1) [42]. Increased β-oxidation and lipolysis are also likely, according to significantly increased mRNA expression of PPARα (peroxisome proliferator receptor α), CPT1α (carnitine palmitoyl transferase 1α) [15,17,42], PGC-1α (PPARγ coactivator 1α) [15,42], fatty acid oxidation metabolism products in the liver and increased ATGL mRNA expression in vivo and in vitro [17,64].

NAR has also been shown to exert hepatoprotective effects in vivo [42,65] that can prevent the development of MAFLD, which is frequently associated with obesity and MetS. Such effects focus on decreasing hepatic enzyme activity and hindering TG and cholesterol deposits, which can be macroscopically and histologically evident [66]. NAR has been shown to increase energy expenditure and regulate autophagy in middle-aged APOE−/−mice by activating AMPK, in addition to inducing a significant decrease in hydroxyproline content in liver and preventing the increase in TGF-ꞵ and α-SMA, potentially reversing hepatic steatosis and preventing liver damage and fibrosis. Furthermore, NAR can significantly increase the expression and activation of SIRT1, a regulator of lipid metabolism, inflammation and oxidative stress, suggesting that some of its beneficial effects could be partly exerted through it [42].

A summary of the in vitro and in vivo research discussed is shown in Table 1.

### 3.5. Body Weight Control

The European, American and Canadian guidelines for the management of obesity agree that after the approach and diagnosis of obesity and its complications, it is necessary to offer individualized treatment focused on lifestyle changes, including dietary pattern and physical activity [63,67,68]. Body weight loss is associated with improvements in multiple biomarkers associated with IR, dyslipidemia, inflammation and OS, as well as changes to the adipokine profile and decreased hepatic steatosis and histological lesions [66,69,70].

NAR has shown a protective effect against weight gain from the early phases of its administration. Ke et al. [15] reported a decrease in body weight in mice after dietary intervention with NAR (3% NAR-supplemented diet) that was associated with a decrease in total adiposity of up to 54%, with a tendency to decrease food intake by approximately 14%, as reported by [17,18]. Burke et al. [17] reported that dietary NAR supplementation significantly prevented weight gain from the first week of treatment; when compared with the control group on a standard diet in LDLR^−/−^ mice; however, these effects were observed in non-obese mice not fed an HFD. This is consistent with Liu et al. [19], where HFD-induced obese rats (12 weeks) had significantly less weight gain when their diet was supplemented with NAR (100 mg/kg), as compared with those fed HFD only. This suggests that the preventive effect against weight gain exerted by NAR may be evident even in already-obese organisms. The reduction in weight gain induced by NAR may be due to a decrease in adiposity and an increase in energy expenditure [17]; however, further investigations are required to elucidate the precise mechanisms.

Murugesen et al. [71] performed a clinical trial in a single diabetic female patient, in which a whole orange extract *(Citrus Sinensis*), equivalent to 150 mg NAR, was administered 3 times a day for 8 weeks along with the usual food intake. The authors reported decreased body weight (2.3 kg), decreased insulinemia (18%) and increased metabolic rate. They, therefore, proposed that NAR is safe in humans. Similarly, Rebello et al. [24] conducted an 18-person, randomized, double-blind, single ascending dose crossover clinical trial to determine the tolerability, safety, and pharmacokinetics of NAR in humans. In this study, capsules containing 536 mg of whole orange extract (*Citrus Sinensis*) were used, which were equivalent to 150 mg NAR. An ascending dose curve was performed with doses of 300, 600 and 900 mg of NAR. It was concluded that doses of 150 to 900 mg of NAR were safe in healthy adults, since no adverse effects or changes in blood markers were reported. However, body weight was not measured in this study.

In addition to NAR, other “citroflavonoids” have also shown health benefits [72], including antiobesity [73]. For example, hesperidin, another main citrus fruit flavanone, has also been found to exert antioxidant, antidiabetic, anti-inflammatory and antiobesity effects [73,74]. Fukuchi et al. [75] used an in vivo model, wherein the animals were fed a high-fat diet containing a lemon peel extract that contained hesperidin, and found a reduction in body weight gain. However, one limitation of this study is that hesperidin was not employed in its pure form; future research about citroflavonoids, in addition to NAR, are, therefore, necessary.

## 4. The Hunger–Satiety Pathways

Long term maintenance of an adequate energy balance and body weight requires controlling energy intake and expenditure. This involves complex and structured neuronal circuits that regulate feeding and metabolism, in response to the integration of peripheral signals from the gastrointestinal tract and adipose tissue [7].

Regulation of the hunger–satiety pathway is mediated in the short and long term by peptides derived from the intestine or enterohormones and adipose tissue, respectively. These molecules can induce either orexigenic (hunger-inducing) or anorexigenic (hunger-inhibiting) effects, when their signaling is integrated by the hypothalamus [76].

There are different central and peripheral mechanisms that simultaneously regulate the hunger–satiety pathway. Food intake is centrally controlled by structures such as the hypothalamus and brain stem. The hypothalamus is an area in the brain made up of multiple nuclei, including the arcuate nucleus (ARC), paraventricular nucleus (PVN), dorsomedial nucleus (DMN), ventromedial nucleus (VMN), and the lateral hypothalamic area (LHA), which integrate and regulate signals in the hunger–satiety pathway. The ARC is critical for the integration of these signals, since it is located in an area that lacks a functional blood–brain barrier, which allows this structure to be exposed to peripheral hormones and peptides [77]. The ARC also sends neuronal projections to the rest of the aforementioned nuclei, regulating the secretion of anorexigenic or orexigenic substances, depending on their function and neuropeptide expression [78]. In this way, the ARC contains two “first order” neuronal populations that, when activated or inhibited, express neuropeptides with opposite effects, either orexigenic or anorexigenic [79]. The AgRP/NPY (agouti-related protein/neuropeptide Y) neuronal population in the ARC stimulates food intake by promoting the expression of orexigenic peptides. Through second-order neurons, this population inhibits the PVN, DMN and VMN, and stimulates LHA which, through the expression of orexin or melanin concentrating hormone (MCH), result in induced food intake. On the contrary, the neuronal population in the ARC induces anorexigenic effects; it is composed of proopiomelanocortin (POMC) neurons that express POMC, α-melanocyte-stimulating hormone (α-MSH), as well as the cocaine and amphetamine-regulated transcript (CART). Through second-order neurons, this population inhibits LHA and stimulates PVN, DMN and VMN to inhibit food intake [78]. Additionally, NPY/AgRP neurons project to POMC neurons and, when stimulated, inhibit and antagonize the anorexigenic effect of the POMC zone [80].

The stem or brainstem also participates in this signaling. It contains the dorso-vagal complex, which consists of the dorsal motor nucleus of the vagus, the area postrema, and the nucleus of the solitary tract (NST). The dorso-vagal complex, in general, receives peripheral signals and transmits them to the hypothalamus, since it contains receptors for circulating signals and peripheral enterohormones [80].

There are other regulatory mechanisms such as the endocannabinoid system [81] and endogenous opioid [82]; however, they will not be considered further in the present work.

Peripheral signals derived from the gastrointestinal tract and adipose tissue relay information to the central nervous system about short-term and stored energy availability. This signaling is mediated by hormones, peptides and changes in gastrointestinal motility, such as accommodation, distension and gastric emptying, which are transmitted by the vagus and spinal nerves to NST, thereby allowing to control the initiation and termination of feeding. The enteroendocrine cells in the gastrointestinal tract basolaterally secrete enterohormones to regulate short-term food consumption; this is carried out in response to nutrients or other molecules contained in the lumen of the small intestine [11]. Some of the most relevant ones include ghrelin, CCK (cholecystokinin), GLP-1 (Glucagon-like Peptide 1), PYY (Peptide Tyrosine-Tyrosine) and insulin; in addition, the adipokines leptin and adiponectin that derive from white adipose tissue (Table 2). The main functions of these molecules and the mechanisms by which NAR could peripherally modulate satiety are described in the following sections.

### 4.1. Ghrelin

Ghrelin is a hunger-inducing (orexigenic) peptide, that is synthesized and secreted primarily in the stomach. In healthy individuals, ghrelin promotes food intake in the short term, that is, circulating levels of ghrelin increase during fasting and decrease after food intake. It also regulates the induction of adiposity in the long term [93].

In the obese state, circulating ghrelin levels have been shown to be dysregulated, either by decreased levels or failure to decrease after food consumption [83,94,95]. This dysregulation has been associated with decreased expression of ghrelin mRNA in the stomach and of its receptor in the hypothalamus; in this way, the activation of agouti-related protein/neuropeptide Y (AgRp/NPY) neurons in the arcuate nucleus (ARC) is suppressed [96,97].

Regarding the effect of NAR on ghrelin, a study in HEK293T cells expressing the ghrelin receptor GHSR indicated that NAR was able to activate it, resulting in a subsequent intracellular mobilization of calcium [84], whereas naringin (the precursor of NAR) showed its prokinetic capacity in an in vivo model [84]. The effect of other phenolic compounds on ghrelin has also been reported. In this regard, [98] showed that a phenolic-rich extract of white grape juice (quercetin, kaempferol, isorhamnetin derivatives, procyanidin, catechin and epicatechin) administered to zebrafish for 4 weeks, reduced their BMI (*p* < 0.001), and decreased ghrelin mRNA in the brain and the intestine (*p* < 0.05), when compared with the obese control. Boix-Castejón et al. [5] also demonstrated that phenolic compounds from hibiscus and lemon verbena could modulate appetite in humans (randomized controlled trial), via decreased ghrelin and increased GLP-1.

To the best of our knowledge, there is minimal information on the effect of NAR on ghrelin, and none of the investigations have used NAR as an isolated compound in an in vivo model, highlighting the need for further research.

### 4.2. Cholecystokinin (CCK)

CCK is a peptide hormone primarily released by enteroendocrine L cells of the duodenum and proximal jejunum, which induces an anorexigenic effect in response to feeding, particularly after meals that contain fat and protein [99]. CCK regulates energy consumption in the short-term through a satiety effect, decreased amount of food and intervals between meals [100]. CCK also exerts effects that contribute to a satiety effect, such as stimulating insulin secretion and delaying gastric emptying [101].

**Figure 3:**
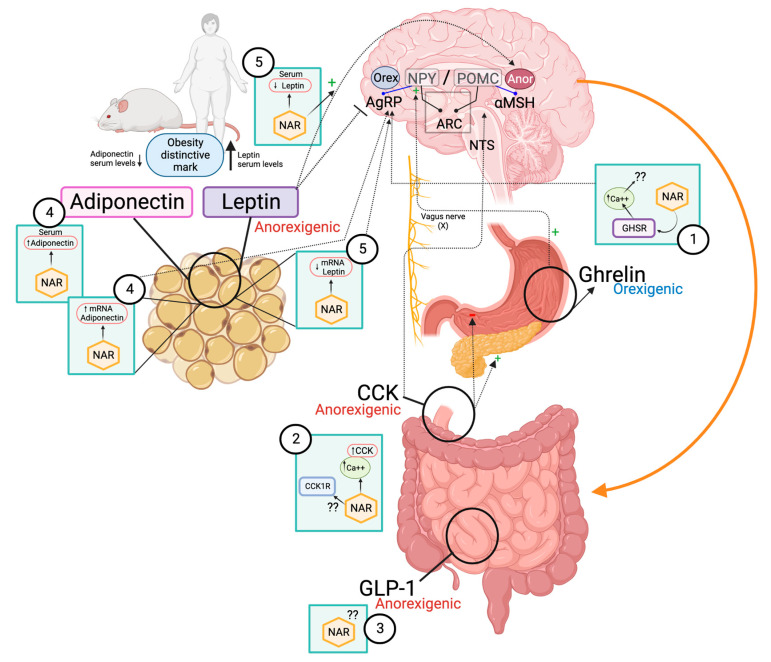
Potential effects of NAR on peripheral pathways of hunger–satiety. (**1**) NAR activates the ghrelin receptor (GHSR) with a subsequent increase in intracellular calcium. (**2**) NAR can dose-dependently increase in vitro CCK secretion by activating the TRPA1 channel and induce calcium signaling. (**3**) GLP-1 is synthesized in the ileum and colon and exerts an anorexigenic effect through vagal terminations, in addition to stimulating insulin secretion and delaying gastric emptying such as CCK; however, the effect of NAR on GLP-1 is unknown. (**4**) NAR prevents hypoadiponectinemia in obesity and increases its expression and that of its receptor (AdipoR2) in adipose tissue. (**5**) NAR decreases circulating leptin in vivo and its expression in adipose tissue, while restoring central sensitivity to it.

It has been reported that an in vitro NAR treatment on STC-1 murine enteroendocrine cells exerted a significant dose-dependent increase in CCK secretion, by a mechanism related to higher intracellular calcium concentrations [87]. The same study also proposes that the effect of NAR on CCK is mediated by activating the TRPA1 channel, which allows the entry of extracellular calcium into the cell, since antagonists of this channel countered the effect of NAR on CCK. This is in agreement with the findings of Feng et al. [102], who reported the blockade of ion channels in ARC neurons after NAR administration. Thus, NAR appears to exert some of its effects by modulating ion channel activity and intracellular calcium signaling (Figure 3).

On the other hand, the ability to modulate CCK secretion by increasing calcium flux has been observed with other flavonoids in vitro. For example, Kim et al. [103] used hesperetin to dose-dependently increase CCK release. Other CCK modulators include quercetin, which has shown a CCK releasing potential, as compared to the glycoside form, rutin [104]. This effect is due to the aglycone form of flavonoids, which usually has stronger activity than its glycoside form. Additionally, what determines the differences in mechanistic aspects between different flavonoids is mainly due to their structure, e.g., the position and number of hydroxyl groups in the aglycone form [104].

In contrast, some phenolic compounds have shown an antagonistic effect on CCK signaling. Shukor et al. [105] reported that tannic acid and gallic acid showed an antagonistic effect on the CCK receptor, with tannic acid also sequestering the CCK-8S peptide, and thus reduced the interaction of the CCK-8S ligand to prevent CCK activation. Further investigations are, therefore, required to determine the mechanism by which NAR (and other phenolics) alters food intake and its effect on the CCK receptor.

### 4.3. Glucagon-like Peptide 1 (GLP-1)

GLP-1 is synthesized and co-secreted with PYY, primarily by L cells of the ileum and colon in response to carbohydrate and fat intake. GLP-1 is considered an incretin-type appetite regulator since, in addition to acting as an anorexigenic peptide by exerting its effects through afferent vagal terminations in the ARC, it mediates pancreatic insulin release, inhibits glucagon and delays gastric emptying [106]. Plasma concentration of GLP-1 is characterized by being low during fasting and increasing with food intake; however, obesity is associated with decreased levels of GLP-1. This is thought to be secondary to a decrease in GLP-1 secretion instead of a dysregulation of its ability to exert an anorexigenic effect at the central level [88].

**Figure 4:**
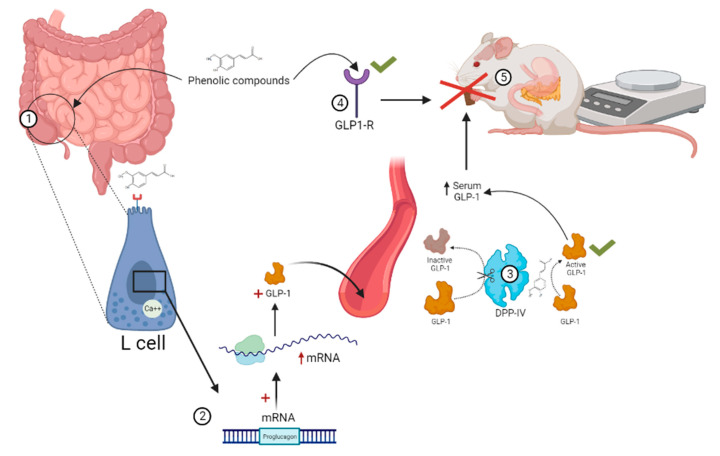
Potential mechanisms by which phenolic compounds can modulate GLP-1. (**1**) The ileum and terminal colon contain the highest density of enteroendocrine L-type cells that synthesize and secrete GLP-1. Epigallocatechin-3-gallate, ferulic acid, genistein, rutin and hispidulin have shown the ability to increase GLP-1 secretion. (**2**) Extracts of phenolic compounds increase mRNA expression of the proglucagon gene (GLP-1 precursor). (**3**) Rutin inhibits the activity of DPP-IV, which inactivates GLP-1, thereby increasing in circulating half-life. (**4**) Puerarin increases mRNA expression of the GLP-1 receptor (GLP-1R) and signaling. (**5**) Increased serum GLP-1 after consuming phenolic compounds results in an anorexigenic effect and suppression of body weight gain in vivo.

Regarding the effect of NAR on GLP-1, no publications were found in the ELSEVIER, PUBMED or GOOGLE SCHOLAR databases when searching for articles using the terms “naringenin and GLP-1” and without specifying date of publication. Therefore, relevant information is presented on the effect of other phenolic compounds that could share similarities with NAR on GLP-1 (Figure 4). In this regard, Zhang and Zhu [107] described some characteristics of flavonoids that allow them to interact with the GLP-1 receptor (GLP-1R) and exert agonist activity, as determined using a density functional theory (DFT) and molecular docking approach. They considered some flavonols (flavone, flavonol, 4′ hydroxyflavonol, 3′, 4′-dihydroxyflavonol and quercetin) and propose that some structural features are key, such as the presence and position of hydroxyl groups, and that, as the number of hydroxyl groups increases, they are able to form more hydrogen bonds with GLP-1R, thereby improving their potential as agonists. This is relevant since GLP-1R agonist drugs are currently indicated for T2DM and weight control, due to the secondary effect of reducing food intake [108], which reflects the therapeutic potential in satiety control.

Moreover, it has been reported that epigallocatechin-3-gallate, ferulic acid, genistein, rutin, puerarin and hispidulin can modulate GLP-1 secretion [109,110,111,112,113,114]. In particular, the flavonoid rutin has been reported to increase GLP-1 secretion in STC-1 cells, while also inhibiting DPP-IV in a dose-dependent manner [109]. Wang et al. [113] used the flavone hispidulin and demonstrated its ability to stimulate GLP-1 secretion in vitro at physiologically relevant concentrations; however, its ability to inhibit DPP-IV was ruled out.

So far, the modulatory effect of GLP-1 induced by phenolic compounds by possibly related to the increase in GLP-1R signaling, inhibitory activity on DPP-IV [109], increased mRNA expression of the proglucagon gene (GLP-1 precursor) and modulation of calcium mobilization [111], however NAR mechanisms are unknown. Further research is, therefore, required to help clarify the mechanism of action, either by altering gene expression, synthesis or secretion of the hormone, and/or decreased activity of DPP-IV.

### 4.4. Peptide Tyrosine-Tyrosine (PYY)

PYY is a member of the pancreatic polypeptide family. It is synthesized and secreted mainly by L-type cells of the distal small intestine and colon [115]. PYY has been associated with delayed gastric emptying [116], which favors its satiety effect. In normal weight subjects, circulating PYY increases after food intake and reaches its maximum concentration after 60–90 min. In obese subjects, the results are controversial, since cases of low PYY levels without a postprandial increase have been identified [89]. As described for other peptides, the secretion and activity of PYY is influenced by the presence of other peptides, exerting a synergistic effect on satiety in the presence of GLP-1.

To the best of our knowledge, there is no information regarding the effect of NAR on PYY. Regarding other phenolic compounds, a single investigation reports how the flavanol epigallocatechin-3-gallate (EGCG) significantly and dose-dependently stimulated the secretion of GLP-1, CCK and PYY in Caco-2 cells. A subsequent ex vivo experiment confirmed the secretion of these anorexigenic hormones in different portions of the intestine [117]. Thus, the effect of NAR on PYY modulation needs to be evaluated.

### 4.5. Adipokines

Signals derived from adipose tissue include feedback mechanisms that are generated in response to food intake and body fat accumulation. These signals regulate the hunger–satiety pathway in the long term, by crossing the blood–brain barrier and acting directly on different populations of neurons in the ARC that stimulate or inhibit food intake [118].

Adipocytes produce and release adipokines (also known as adipocytokines), hormones that are structurally similar to cytokines, with anti- or pro-inflammatory properties. They initially exert an anti-lipotoxic function in order to protect other tissues from the cytotoxic effects of high concentrations of fatty acids, as well as a feedback function in the hunger–satiety pathway [119]. However, in obesity, abdominal adipose tissue dysfunction has been identified due to adipocyte hypertrophy, increased macrophage infiltration, and dysregulation of adipokine secretion and concentrations, with an imbalance in favor of a proinflammatory state that contributes to metabolic disorders and systemic inflammation [120]. These adipokines are involved in the regulation of systemic processes such as food intake, nutrient metabolism, insulin sensitivity and inflammation [49]. Adiponectin and leptin are the more studied adipokines.

#### 4.5.1. Adiponectin

Adiponectin is a polypeptide hormone, produced and secreted mainly by adipocytes. Plasma adiponectin inversely correlates with adiposity and BMI, whereas hypoadiponectinemia has been reported in the obese, and metabolic disorders such as MetS, IR and T2DM [91]. In contrast, plasma adiponectin increases after food restriction and weight loss, as well as by certain dietary components [121]. This adipokine has shown an ability to lower body weight and plasma lipids, as well as synergy with insulin to suppress gluconeogenesis [122]. Adiponectin is categorized as anti-inflammatory, since it inhibits the production of TNF-α and IL-6 by inhibiting the activation of NF-ĸB and by stimulating the release of IL-10 [123]. It is also considered to be antidiabetic, since it sensitizes cells to insulin signaling [122] and promotes fatty acid oxidation [124].

Regarding the effects of NAR on adiponectin, an intervention model study demonstrated that doses of 50 and 100 mg prevented the decrease in its serum concentration in rats fed an HFD (*p* < 0.05) [19]. Likewise, NAR has been associated with increased expression of adiponectin mRNA in cultured human adipocytes (*p* < 0.01) [64], 3T3-L1 cells [125] and in vivo [92]. An in vitro study reported an increase in the expression of adipoR2 in T3-L1 adipocytes after exposure to NAR chalcone [126]. In contrast, Ke et al. [15] did not identify significant changes in adiponectin expression or concentration in mice fed a NAR-supplemented diet. However, various studies suggest that NAR may exert a protective effect by modulating circulating adiponectin and its mRNA expression.

Naringin, NAR’s precursor, has also been studied in humans. For example, Barajas-Vega et al. [127] reported a double-blind, placebo-controlled, randomized clinical trial in 14 adults with previously diagnosed dyslipidemia, that received 450 mg of naringin/day for a 90-day period. They report significantly decreased BMI, as well as positive lipid profile changes (decreased total and LDL cholesterol) and increased adiponectin, which could suggest a potential therapeutic use of NAR’s precursor.

#### 4.5.2. Leptin

Leptin is a product of the obese gene (*ob*). It is a hormone synthesized and secreted mainly by adipocytes [90], whose effects are anorexigenic [128]. Leptin decreases AgRP/NPY expression and stimulates proopiomelanocortin (POMC) [129], showing an inhibitory effect on food intake and body weight. It also decreases gastric emptying by regulating sympathetic nerve activity [130], which has been associated with a decrease in the amount of food ingested, but not with changes in feeding frequency [131]. Circulating leptin is elevated in obese subjects (hyperleptinemia); however, such an increase is not related to decreased food intake [90]. This is due to a decrease in central sensitivity to leptin, which is why obese subjects are considered resistant to leptin and its anorexigenic effects. There are various mechanisms that contribute to this resistant state, for example, a decrease in the transport of serum leptin to the hypothalamus, suppression of the leptin signaling pathway (specifically, the long LepRb receptor), which becomes evident due to the decrease in STAT3 phosphorylation, endoplasmic reticulum stress and the response to unfolded proteins [132].

NAR has shown the ability to modulate leptin. In this regard, dietary NAR supplementation (3% wt/wt) in a mouse model decreased fasting serum leptin by up to 80%, and expression in adipose tissue from 55 to 60% [15]. These results coincide with those of other authors [19], and strongly correlate with the decrease in obesity. Related to this effect, NAR can also restore central leptin sensitivity and its anorexigenic actions, for example, decreased leptin was associated with increased STAT3 phosphorylation in the hypothalamus, which suggests that NAR restored leptin sensitivity after an obesogenic diet [19,132]. It is worth mentioning that NAR has been shown to maintain its efficacy in leptin-deficient models (such as ob/ob mice); therefore, its effect on leptin regarding satiety would not be the only mechanism that can modulate food intake [17,133].

Most in vivo and in vitro studies describe a dose-dependent effect of NAR [19,42,87], still, its use has been studied in vivo at low doses (25 mg/kg body weight) [19] in a short period of time (2 days, intraperitoneal) [102] with positive results (see Table 1), which may support its use as a dietary supplement [65]. It is also important to consider that the documented effects regarding NAR are mainly preventive, protective and related to an improvement, but are not curative. This makes it evident that there is a need for further research, including clinical trials to verify the effects of NAR on body weight control and mediators involved in the hunger–satiety pathway (peptides and peripheral hormones).

## 5. Conclusions

NAR is a bioactive compound with various bioactivities. The evidence suggests that it may modulate multiple pathways involved in the onset and progression of obesity and its comorbidities, including IR, inflammation, OS, macrophage infiltration, dyslipidemia, hepatic steatosis, decreased adiposity and weight gain. NAR is also capable of modulating some peptides directly associated with the hunger–satiety pathway, such as ghrelin, cholecystokinin, insulin, adiponectin, and leptin. NAR supplementation could be implemented to prevent weight gain, but continued research is still required.

## Figures and Tables

**Table 1:** Findings from in vivo and in vitro studies evaluating metabolic effects of naringenin (NAR) treatments.

Experimental Model (Reference)	Dose and Route of Administration	Time	Effects	Findings
C57BL/6J obese male mice, HFD (60% calories from fats) [50]	100 mg/kg/day, P.O.	14 days	Antiinflammatory	NAR suppresses neutrophil infiltration in adipose tissue secondary to an HFD (*p* < 0.05 for HFD vs. HFD + NAR). Decreasing trend in the expression of MCP-1, IL-6, MIP-1α, MIP-2 and significant decrease in MCP-3 in adipose tissue (*p* < 0.05 for HFD vs. HFD + NAR).
Female mice with gestational diabetes, heterozygotes B6.BKS(D)-Lepr ᵈᵇ/⁺/J [45]	50 mg/kg dissolved in dimethyl sulfoxide (DMSO), I.P.	8 days	Antihyperglycemic	NAR lowers fasting glycemia by 15% (*p* = 0.0127 vs. control).
			Antiinflammatory	Significant decrease in IL-1A mRNA expression in visceral adipose tissue (*p* < 0.05 vs. control).
			Antioxidant	NAR increased GR mRNA expression (*p* < 0.05 vs. control), as well as decreased CAT mRNA expression (*p* < 0.05 vs. control) in visceral adipose tissue. NAR increased mRNA expression of SOD1 (*p* < 0.05 vs. control) in subcutaneous adipose tissue.
C57BLKsJ db/+ (db/+) mice. Standard diet (29% protein, 47% carbohydrates, 17% fats) [16]	100 mg/kg/bw/day Oral gavage, 1% CMC	4 weeks	Antihyperglycemic	NAR lowered glycemia (0 min *p* < 0.05 GDM vs. GDM + NAR; 30 min *p* < 0.05 GDM vs. GDM + NAR; 60 min *p* < 0.05 GDM vs. GDM + NAR; 90 min *p* < 0.01 GDM vs. GDM + NAR; 120 min *p* < 0.01 GDM vs. GDM + NAR) (fasting glycemia 6.84 ± 1.03 mmol/L GDM vs. 4.38 ± 0.89 mmol/L GDM + NAR, *p* < 0.05), improved glycemic profile, including HOMA-IR (9.52 ± 0.31 GDM vs. 6.12 ± 0.23 GDM + NAR, *p* < 0.05), but without normalizing to the parental strain.
			Antiinflammatory	Significant decrease in proinflammatory cytokines in serum and skeletal muscle (GDM vs. GDM + NAR), IL-1β (*p* < 0.05 serum, *p* < 0.01 skeletal m.), IL-6 (*p* < 0.01 serum and skeletal m.), TNF-α (*p* < 0.01 serum and skeletal m.) and MCP-1 (*p* < 0.01 serum, *p* < 0.05 skeletal m.).
			Antiobesogenic	Significantly lower body weight gain, without weight normalization when compared to the parental strain (*p* < 0.05 for GDM vs. GDM + NAR).
In vitro, C2C12 mouse myoblasts [16]	50 μg/mL NAR	-	Antihyperglycemic	Increased AMPK-dependent membrane translocation of GLUT4.
			Antioxidant	NAR decreases ROS levels in C2C12 cells treated with TNF-α, in an AMPK-dependent manner.
In vivo, male LDLR^−/−^ mice. Rodent chow (12% of calories from fat, 16% protein; isocaloric diet) [17]	NAR in diet 3% wt/wt (supplementation). P.O.	8 weeks	Antihyperglycemic	NAR decreased fasting glycemia by 37% (*p* < 0.05 for chow vs. chow + NAR), fasting insulinemia by 57% (*p* < 0.05 for chow vs. chow + NAR) and improved HOMA-IR (*p* < 0.05 for chow vs. chow + NAR).
			Antihyperlipidemic	Decreased levels of TG and TC (46% and 23%, respectively; *p* < 0.05 for chow vs. chow + NAR). Increased fatty acid oxidation in the liver through increased serum levels of ꞵ-hydroxybutyrate (33%; *p* < 0.05 for chow vs. chow + NAR) and increased expression of hepatic genes involved in fatty acid oxidation (PGC-1a, 47%, *p* < 0.05 for chow vs. chow + NAR; Cpt1a, 15%, trend) and lipolysis (Pnpla2(ATGL) 33%, *p* < 0.05 for chow vs. chow + NAR).
			Antiobesogenic Satiety	Reduced in weight gain (~8–10%; *p* < 0.05 for chow vs. chow + NAR) by decreased adiposity (eWAT (69%) and iWAT (71%), (*p* < 0.05 for chow vs. chow +NAR) and increased energy expenditure. No significant effects on satiety.
			Antihyperglycemic	Reversed insulinemia by 50%. Decreased fasting glycemia (trend-13%). Improved HOMA-IR.
Obese LDLR^−/−^ male mice. High fat and cholesterol diet (HFHC) (42% of calories from fat, 0.2% cholesterol) [18]	NAR 3% wt/wt dietary supplementation. P.O.	12 weeks	Antiinflammatory	Decreased mRNA expression of TNF-α, Ccl2 and Ccl3 (trend).
			Antihyperlipidemic	Reduced total cholesterol (TC) and TG by ˃50% (*p* < 0.05 HFHC vs. HFHC + NAR)
			Antiobesogenic	Decreased adipose tissue hypertrophy with decreased epididymal adipocyte area by 19% (*p* < 0.05 HFHC vs. HFHC + NAR) and epididymal tissue reduction by 29% (*p* < 0.05 HFHC vs. HFHC + NAR). Induced body weight loss of ~13% after intervention (*p* < 0.05).
			Hepatoprotective	Reversed intrahepatic TG at the end of the experimental period (58–82%) (*p* < 0.05 HFHC vs. HFHC + NAR). Reversed suppression of genes involved in β-oxidation, increasing its expression up to 1.4× (Cpt1α) and reduces (trend) Srebp1c, suggesting reduction in de novo lipogenesis.
			Satiety	Intervention with NAR + HFHC showed no significant changes in food intake between groups. An aversion to NAR taste initially documented, which decreased slowly increasing the dose of the flavonoid in the first week to prevent a significant impact on food intake.
			Antihyperglycemic	Decreased fasting glycemia at week 18 (163.0 ± 5.2 mg/dL vs. 127.1 ± 10.2 mg/dL; *p* < 0.05 CT vs. NAR) and HOMA-IR (*p* < 0.05 CT vs. NAR).
In vivo, intervention model. Ovariectomized female C57BL/6J mice. Semi-purified diet (control) protein 20% kcal, carbohydrates 70%, fats 10% [15]	NAR 3% wt/wt	11 weeks (after 11 weeks of induction)	Antiinflammatory	Decreased mRNA expression of MCP1 (56%) and IL-6 (40%) in perigonadal adipose tissue (*p* < 0.05 CT vs. NAR).
			Antihyperlipidemic	Decreased serum TC (*p* < 0.05 CT vs. NAR), evident by H&E staining. Increased mRNA expression of Srebp1, Cpt1α, PGC1α (4-fold and PEPCK (3.5-fold) (*p* < 0.05 CT vs. NAR).
			Hepatoprotective	Decreased total lipids and TG in the liver (*p* < 0.05 CT vs. NAR)
			Antiobesogenic	Decreased body weight (*p* < 0.05 CT vs. NAR). Decrease in total adiposity (intra-abdominal and subcutaneous) of 54, 59 and 50%, respectively (*p* < 0.05 CT vs. NAR).
			Satiety	Decreased leptin by 80% (*p* < 0.05 CT vs. NAR). Decreased caloric intake (~14%; week 12, *p* < 0.05 CT vs. NAR; week 13–22, trend, *p* = 0.075).
			Antilipidemic	Increased gene expression associated with thermogenesis and fat oxidation: UCP1, PGC-1α and PGC-1β, ATGL, CPT1β (*p* < 0.01, CT vs. NAR).
In vitro, human white adipocyte culture (hADSC) and abdominal subcutaneous white adipose tissue (pWAT) from human subjects. [64]	8 μM	7 days for hADSC	Antihyperglycemic	Increased mRNA expression of GLUT4, ChREBP α + β, adiponectin (*p* < 0.01, CT vs. NAR).
			Antidyslipidemic	Significantly decreases TG, TC and LDL (*p* < 0.05, HFD vs. NAR; 50 and 100 mg/kg) Increases HDL-c (*p* < 0.05, HFD vs. NAR; 50 and 100 mg/kg)
Male Wistar rats. High-fat diet (22% protein, 27% fats, 41% carbohydrates) [19]	NAR 25,50 and 100 mg/kg, dissolved in DMSO	4 weeks	Antioxidant	Significant decrease in plasma MDA and NO (*p* < 0.05, HFD vs. NAR; 50 and 100 mg/kg) (partial prevention in the increase in MDA). Significant increase in SOD and GSH (*p* < 0.05, HFD vs. NAR; 50 and 100 mg/kg).
			Antiobesogenic	Decreased weight gain (*p* < 0.05, HFD vs. NAR 100 mg/kg). Decreased epididymal and visceral adipose tissue (*p* < 0.05, HFD vs. NAR; 50 and 100 mg/kg)
			Hormonal responses	Prevented increased plasma leptin significantly (*p* < 0.05, HFD vs. NAR; 50 and 100 mg/kg) Prevented decreased adiponectin in HFD (*p* < 0.05, HFD vs. NAR; 50 and 100 mg/kg) Increased hypothalamic STAT3 phosphorylation (*p* < 0.05, HFD vs. NAR; 50 and 100 mg/kg)

HFD = High-fat Diet; NAR = Naringenin; P.O. = oral intake; I.P = intraperitoneal; MCP-1 = Monocyte chemoattractant protein-1; CCL2 = Chemokine (CC-motif) ligand 2; MCP-3 = Monocyte chemoattractant protein-3; MIP-1α = Macrophage inflammatory protein-1 alpha; CCL3 = Chemokine (CC-motif) ligand 3; MIP-2 = Macrophage inflammatory protein-2; IL-6 = Interleukin 6; DMSO = Dimethyl sulfoxide; IL-1A = Interleukin 1A; GR = Glutathione reductase; CAT = catalase; SOD = superoxide dismutase; CMC=Carboxymethyl Cellulose, GDM=Gestational Diabetes Mellitus; HOMA-IR=Homeostatic Model Assessment for Insulin Resistance, IL-1β = Interleukin 1β; IL-6 = Interleukin 6; TNF-α = Tumor necrosis factor alpha; GLUT4 = glucose transporter type 4; AMPK = AMP-activated protein kinase; ROS = reactive oxygen species; PGC-1α = Peroxisome proliferator-activated receptor alpha coactivator; PGC-1β = Peroxisome proliferator-activated receptor beta coactivator; Cpt1α = Carnitine palmitoyltransferase 1-alpha; Cpt1β = Carnitine palmitoyltransferase 1-beta; ATGL = adipose triglyceride lipase; eWAT = epididymal white adipose tissue; iWAT = inguinal white adipose tissue; Srebp1c = transcription factor sterol regulatory element binding protein-1c; PEPCK = Phosphoenolpyruvate carboxykinase; UCP1 = uncoupling protein 1; ChREBP α/β = carbohydrate response element binding protein; TG = triglycerides; TC = total cholesterol; LDL = low density lipoprotein; MDA = malondialdehyde; GSH = glutathione; NO = nitric oxide; STAT3 = signal transducer and activator of transcription 3.

**Table 2:** Main enterohormones involved in the hunger–satiety pathway, and findings from in vivo and in vitro studies evaluating metabolic effects of naringenin (NAR) treatments.

Hormone/Physiological Effect	Entero-Endocrine Cell Type	Localization by Highest Density	Main Receptor in Hunger–Satiety Pathway	Levels in Normal Weight	Levels in Obesity	Reported Effects of NAR
Ghrelin Orexigenic	P	Stomach	GHSR1A	Increase before meals, decrease after meals.	Low before meals and shorter duration of suppression after meals [83]	Ghrelin receptor is activated by NAR in vitro [84]
CCK Anorexigenic	I	Duodenum and proximal jejunum	CCK1	Increase after meals, max. concentration 15 min.	Low after meals or failure to decrease after meals [85,86]	NAR stimulates CCK secretion in vitro [87]
GLP-1 Anorexigenic	L	Duodenum and colon	GLP-1	Low before meals, high after meals.	Low before and after meals [88]	NR
PYY (3–36) Anorexigenic	L	Colon	Y2	Increase after meals, max concentration 60–90 min	Variable. Some report low after meals [89]	NR
Insulin Anorexigenic	Pancreatic β-cells	Pancreas	IR	Low (compared with obese state)	High (in the insulin resistant state)	NAR enhances glucose-stimulated insulin secretion and glucose sensitivity in vitro [58] NAR improves insulin sensitivity, improves glucose and insulin tolerance in vivo through AMPK GLUT4 translocation [16]
Leptin Anorexigenic	Adipocyte	Adipose tissue	LepRb	Low (compared with obese state)	High [90]	NAR decreases serum leptin [19] and it′s expression in vivo [15]
Adiponectin Anorexigenic	Adipocyte	Adipose tissue	AdipoR1 and adipoR2	High (compared with obese state)	Low (inversely proportional to adipose tissue mass) [91]	NAR increases serum adiponectin levels despite an HFD in vivo [19]. NAR enhances adiponectin mRNA expression in vivo [92]

NAR = naringenin; CCK = cholecystokinin; GLP-1 = glucagon-like peptide 1; PYY = peptide YY; mRNA = messenger RNA; NR = not reported.

## Data Availability

Not applicable.
